# Quality of life and associated factors among people with epilepsy in Ethiopia: a systematic review and meta-analysis

**DOI:** 10.1186/s12889-024-19018-3

**Published:** 2024-06-07

**Authors:** Fantahun Andualem, Mamaru Melkam, Gebrieslassie Tadesse, Girum Nakie, Techilo Tinsae, Setegn Fentahun, Gidey Rtbey, Girmaw Medfu Takelle, Berihun Agegn Mengistie, Getachew Muluye Gedef

**Affiliations:** 1 Department of Psychiatry, College of Medicine and Health Science, University of Gondar, PO Box 196, Gondar, Ethiopia; 2 Department of General Midwifery, College of Medicine and Health Science, University of Gondar, Gondar, Ethiopia; 3 Department of Psychiatry, School of Medicine, College of Medicine and Health Science, University of Gondar, Gondar, Ethiopia

**Keywords:** Prevalence, Quality of life, Associated factors, People with epilepsy, Patients with epilepsy, Ethiopia

## Abstract

**Background:**

Epilepsy is a global health and economic burden with major problems that have an impact on physical, psychological, and social activities. Quality of life (QoL) is often disturbed and can be influenced by many factors, like anti-seizure medication side effects, the sociocultural environment, and various disease-related factors. The aim of this systematic review and meta-analysis is to provide an overview of the most recent information available regarding the pooled prevalence of poor quality of life and associated factors among adult people with epilepsy in Ethiopia.

**Methods:**

The Preferred Reporting Items for Systematic Reviews and Meta-Analyses (PRISMA) is an appropriate set of guidelines for reporting systematic reviews and meta-analyses. This systematic review and meta-analysis protocol was registered on the International Prospective Register of Systematic Reviews (PROSPERO) with CRD42024527914. To find publications for the systematic review and meta-analysis, we used both manual and electronic searches. The publications were searched by PubMed, MEDLINE, EMBASE, Cochrane Library, Scopus, and other grey publications were searched by Google Scholar. The Joanna Briggs Institute (JBI) for cross-sectional study quality assessment was employed to evaluate the methodological quality of the studies included in this review. The data was extracted in Microsoft Excel, and then it was exported into STATA 11.0 for analysis. A funnel plot and an objective examination of Egger’s regression test were used to check for publication bias.

**Results:**

We have included 7 studies conducted in Ethiopia with 2123 study participants, of whom 1163 (54.78%) were male individuals, and 1196 (56.34%) of the participants were living without marriage (either single, divorced, or widowed). The pooled prevalence of poor quality of life among people with epilepsy in Ethiopia is 45.07 (95% CI: 39.73–50.42%). Further, in subgroup analysis regarding the assessment tool of poor quality of life of people with epilepsy, QOLIE-31 accounted for 50.05% (95%CI: 46.65–53.45) and WHO QOL BREF accounted for 39.72% (95%CI: 27.67–51.78). Among the associated factors, being unable to read and write, anxiey and depression were significantly linked to the quality of life of people with epilepsy.

**Conclusion:**

This review found that there was a high pooled prevalence of poor quality of life related to people with epilepsy in Ethiopia. This study may provide further information to concerned bodies that do early screening and manage the quality of life of individuals with epilepsy. Also, screening and intervention for anxiety and depression problems should be considered in regular epilepsy care management.

**Supplementary Information:**

The online version contains supplementary material available at 10.1186/s12889-024-19018-3.

## Introduction

Recurrent seizures are the hallmark of epilepsy, the most prevalent chronic neurological condition in the general population [1, 2]. A spontaneous, excessive firing of neurons from the brain causes a temporary paroxysmal pathological impairment of cerebral function known as a seizure [1]. Over 70 million individuals globally suffer from epilepsy, with 80% of people with epilepsy (PWE) residing in developing countries [3]. Epilepsy is a major source of mortality and morbidity on a global scale [4]. According to estimates, the age-standardised disability-adjusted life years per 100,000 people were 201.2 for men and 182.6 for women [4]. In comparison to high-income countries, the African area and countries with a low socio-demographic index have been found to have higher age-standardised disability-adjusted life-years (DALYs) and the standardised mortality ratio (SMR) [4, 5]. According to the review, in the lower socioeconomic categories, there appears to be a correlation between the prevalence of epilepsy and socioeconomic status [6].

Epilepsy is a global health and economic burden with major problems that have an impact on physical, psychological, and social activities [7]. Dealing with the difficulties of the disease remains a considerable challenge for PWE, as well as their relatives [8]. Quality of life (QoL) is often disturbed and can be influenced by many factors, like anti-seizure medication side effects, the sociocultural environment, and various disease-related factors [9–11]. Furthermore, epilepsy is surrounded by a number of mental illnesses (anxiety, depression) and social issues that make managing it more difficult [12] and have a substantial negative impact on quality of life [13].

There are several perspectives on how to define quality of life [14]. Certain approaches are based on subjective well-being, phenomenological viewpoints, personal needs, and expectations [15]. A different school of thought on well-being makes an effort to differentiate between methods based on lists of objectives, hedonism, satisfaction of preferences, and life satisfaction [16]. A few definitions of quality of life include “an individual’s perception of their position in life in the context of the culture and value systems in which they live and in relation to their goals, expectations, standards, and concerns [17] and a conscious cognitive judgment of satisfaction with one’s life. While the majority of these definitions of quality of life concentrate primarily on people’s subjective assessments, others have made a compelling case for the inclusion of objective criteria in the definition of quality of life [18–20]. A personal set of values weighs both subjective and objective assessments of one’s physical, material, social, and emotional well-being, as well as one’s level of personal growth and meaningful activity. This is how QoL is defined, for instance [19].

According to the study, 38.6% of PWE in Lebanon had poor quality of life [21]. In the study conducted in an African country, the good quality of life for individuals with epilepsy (49.90%) was lower than that of the normal control group (77.60%) [22]. However, there has been no review of the quality of life among people with epilepsy in Ethiopia. Indeed, the quality of life status of people living with epilepsy has been the topic of a large number of studies, with a large variation in reported prevalence rates from 24.4% [23] to 51% [24]. Based on the review, the aspects that affected patients with epilepsy the most were energy and fatigue. The psychiatry comorbidity of depression was a moderate predictor of QoL, while seizure frequency was a high predictor [25]. Another study stated that poor quality of life was predicted by sociodemographic traits, the existence of psychiatric disorders, and a prolonged history of epilepsy [26].

The results of a systematic review and meta-analysis revealed that depression and anxiety are the most common comorbid mental health conditions in low- and middle-income countries [27]. According to a different study, having mental comorbidities and having little awareness about epilepsy are significant predictors of poor quality of life in PWE [21]. In Ethiopia, 43.8% of individuals with epilepsy reported having depression [28]. According to a systematic review and meta-analysis conducted in Africa, 45.93% of individuals with mental illness reported having poor quality of life. In a subgroup analysis conducted by the county, Ethiopia had a higher prevalence of poor quality of life (48.09%). Each domain of disorders showed a higher frequency of poor quality of life: depressive disorders (38.90%), schizophrenia (48.53%), and bipolar disorder (69.63%) [29]. As a result, people with epilepsy may find it challenging to live a quality life due to the simultaneous effects of concomitant psychiatric problems. Our search revealed that, among Ethiopian quality of life of people with epilepsy, there hasn’t been a comprehensive study or meta-analysis of the prevalence of quality of life of people with epilepsy and its associated factors. Therefore, the aim of this systematic review and meta-analysis is to provide an overview of the most recent information available regarding the pooled prevalence of poor quality of life among adult people with epilepsy in Ethiopia. To close this research gap, the following questions will be addressed:

1. What is the pooled prevalence of quality of life among people with epilepsy in Ethiopia?
2. What are the associated factors of quality of life among people with epilepsy in Ethiopia?

## Materials and methods

### Protocol

The Preferred Reporting Items for Systematic Reviews and Meta-Analyses (PRISMA) [30] is an appropriate set of guidelines for reporting systematic reviews and meta-analyses (Supplementary File 1). This systematic review and meta-analysis protocol was registered on the International Prospective Register of Systematic Reviews (PROSPERO) with CRD42024527914. The guidelines for the meta-analysis of observational studies in epidemiology were followed [31].

### Eligibility criteria

**Fig. 1:**
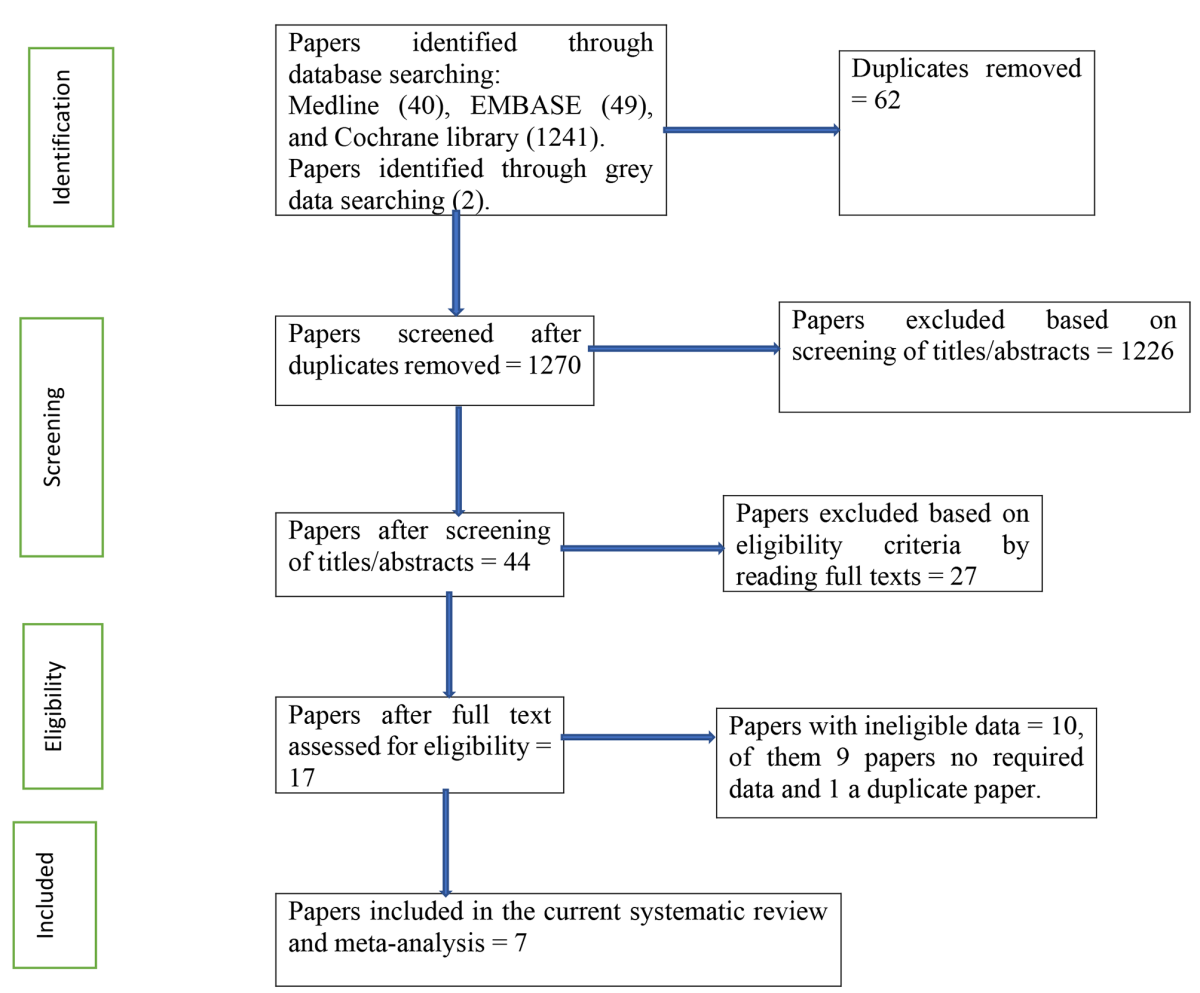
PRISMA flowchart of a review search on the prevalence and associated factors of quality of life among people with epilepsy

Peer-reviewed publications that reported the prevalence and/or factors associated with quality of life of PWE in Ethiopia. The publications were all pertinent observational studies (cross-sectional). To be considered, papers had to be published online in a peer-reviewed journal in English between January 2000 and March 2024, depending on the publication year. However, we did not include the publications that reported the prevalence and/or factors associated with quality of life of PWE in Ethiopia when they reported conference abstracts, duplicates, reviews, commentaries, grey papers, or studies that were not completely accessible. Before including the abstract and title of each paper in our meta-analysis, we reviewed them. After relevant research was chosen, the complete material was reviewed. As can be seen in Fig. 1, we did not include any articles in our study that did not meet the inclusion criteria.

### Sources and data search strategy

The CoCoPop (Condition, Context, Population) technique was used to design the search strategy for this systematic review in the manner described below [32]: The Co (ondition/disease of interest of the study) consisted of QoL among Ethiopians of individuals with epilepsy. Co (context) is the background setting of the condition being studied; it was considered in a clinical setting; and Pop (population) was focused on people with epilepsy. To find publications for the systematic review and meta-analysis, we used both manual and electronic searches. The publications were searched by PubMed, MEDLINE, EMBASE, Cochrane Library, Scopus, and other grey publications were searched by Google Scholar. We used the following free-text keywords (prevalence OR epidemiology OR magnitude) AND (“quality of life” OR QoL) AND (“associated factors” OR predictors) AND (“people with epilepsy” OR “patients with epilepsy”) AND (Ethiopia) (Supplementary File 2). The search was conducted on March 31, 2024.

### Study screening and selection

The two authors, Fantahun Andualem (FA) and Girum Nakie (GN), independently conducted the searching, screening, and selection of the articles. The first step involved importing research papers from the designated databases into EndNote X20 and eliminating duplicates. After excluding irrelevant articles by assessing their title and abstract, the full texts of the articles were read. When studies were located in databases but the information was incomplete, the assigned authors (FA and GN) to data extraction contacted the corresponding author by email to request more information. A cross-check was done by two authors (FA and GN) following searches. If contrasting results occurred between the two authors during the searching, screening, and selection of the articles, we were discussing ways to achieve consensus with other authors.

### Quality assessment

The Joanna Briggs Institute (JBI) for cross-sectional study quality assessment was employed to evaluate the methodological quality of the studies included in this review [33]. Using JBI, the two authors (FA and GN) separately assessed the original research’s quality. Papers that scored five or higher on a nine-point scale were included in this review for analysis. The instrument has a total of nine ratings.

### Data extraction

After the studies that met the eligibility criteria and quality of assessment scores of five or more were found, the data was extracted using a Microsoft Excel file that was preformatted. The two authors (FA and GN) independently extracted all the necessary data from the articles using a standardized data extraction format. The data extraction format included the following items: The first author’s name, the publication year, the region in which the study was conducted, the study design, the quality of life assessment tool, the mean age of the participants, the prevalence of male participants, the prevalence of non-married (either single, divorced, or widowed) participants, the sample size of the participants, the prevalence of quality of life, and associated factors were all included in the data extraction format.

### Data analysis and publication bias

FA computed the logarithm and standard error of the logarithm of the prevalence to investigate the prevalence of poor quality of life among the included studies. Regarding associated factors, odds ratios, the logarithm of the odds ratio, and the standard error of the logarithms of the odds ratio were calculated. The data was extracted in Microsoft Excel, and then it was exported into STATA 11.0 for analysis. Summaries of the data were displayed using the random analysis effects model, and the Q and I^2^ tests were employed to look at study heterogeneity [34]. The low, moderate, substantial, and high heterogeneities were denoted by the ≤ 25%, 25–50%, 50–75%, and ≥ 75% I^2^ heterogeneity thresholds, respectively [35, 36]. Meta-analysis and narrative analysis were used to describe the results. The pooled prevalence was described using percentages and a 95% confidence interval as summary statistics. Sensitivity analysis was used to check whether the overall finding was robust to potentially influential decisions. An estimate of random variation across studies, which is the foundation of the random effects model, was used. A funnel plot [37] and an objective examination of Egger’s regression test [38] were used to check for publication bias. If the Egger’s regression assumption test result was statistically significant (*p* < 0.05) or the funnel plot was asymmetrical, publication bias was reported [37, 38]. The study country, the mental illness of care recipients, the participant domain, the study design, the study setting, and the assessment tool were all the subjects of a subgroup analysis.

## Results

### Description of included studies

There were 1330 publications identified in database searches, and another 2 papers were added through grey searches. Among them, 62 papers were removed due to duplicates; 1226 papers were excluded based on screening of titles or abstracts; 27 papers were excluded based on eligibility criteria by reading full texts; and 10 papers were excluded of them due to a lack of required information [39–47] and a duplicate paper [48]. Finally, a total of 7 studies were involved in this systematic review and meta-analysis (Fig. 1).

### Study quality assessment

The articles’ quality was assessed using the Joanna Briggs Institute’s (JBI) quality evaluation standards. Every article included in this study has good quality and a JBI score of ≥ 5 (Supplementary File 3).

### Characteristics of included studies

We have included 7 studies conducted in Ethiopia with 2123 study participants, of whom 1163 (54.78%) were male individuals. Regarding marital status, 1196 (56.34%) of the participants were living non-marriage (either single, divorced, or widowed). Seven studies were selected, four of which were carried out in Amara reginal state [24, 44, 49, 50], one each in Oromia Regional State [23], South Nation Nationality Regional State [51] and Addis Ababa City [52]. Based on the assessment tool, three studies were assessed using the Quality of Life in Epilepsy (QOLIE)-31questionnaire [53], three studies were assessed using the World Health Organisation Quality of Life questionnaire (WHO QOL BREF) scale [54], and one study was assessed using the Quality of Life in Epilepsy (QOLIE)-10questionnaire [55] (Table 1).

**Table 1:** Characteristics of included studies on quality of life of people with epilepsy in Ethiopia

First author name, publication year	Study region	Study design	Tool	MA (year)	NM (%)	NM (%)	SZ	*P*, %	AFW: AOR (95% CI)
Ayelign MK, 2021 [44]	Amahara	Cross-sectional	QOLIE-31	32.39	164(41.5)	208(52.7)	395	49.6	Male sex: 11.8(1.11, 22.48); Higher educational status: 19.52(3.78, 35.34)
Esileman AM, 2020 [49]	Amahara	Cross-sectional	QOLIE-10	29.1	216(61)	265(74.9)	354	45.2	…
Fentahun M, 2022 [50]	Amahara	Cross-sectional	WHO QOL BREF	28	243(60.4)	190(47.3)	402	47.8	Unable to read and write: 2.51(1.19, 5.28); Able to read and write: 3.11(1.35, 7.15); Medium drug adherence: 8.36(2.41, 28.98); Low drug adherence: 14.65(4.35, 49.38); Anxiety: 3.63(2.55, 8.42); Depression: 3.86(2.16, 6.82)
Gosaye MT, 2020 [23]	Oromia	Cross-sectional	WHO QOL BREF	32	67(55.4)	61(52.1)	121	24.4	…
Minale TT, 2014 [52]	Addis Ababa	Cross-sectional	WHO QOL BREF	28	229(55.2)	269(64.8)	415	45.8	Unable to read and write: 4.69(1.38, 15.87); Primary school: 3.51(1.57, 7.82); Taking two or more drugs: 1.81(1.02, 3.22); Anxiety: 4.49(2.39, 8.44); Depression: 6.62(4.86, 19.05); Perceived stigma: 2.14(1.24, 3.67)
Wudu Y, 2024 [51]	South Nation Nationality	Cross-sectional	QOLIE-31	31.26	183(53.8)	151(44.4)	340	50.3	…
Yonas T, 2022 [24]	Amahara	Cross-sectional	QOLIE-31	28.9	61(63.5)	52(54.2)	96	51	…

Note: *MA: mean age of participants; NM: number of male participants; NM: number of non-marriage (either single, divorced, or widowed) participants; SZ: sample size; P: prevalence of poor QoL of the participants; FAW: factors associated with; AOR: adjusted odd ratio; CI: confidence interval*

### The pooled prevalence of quality of life of people with epilepsy

**Fig. 2:**
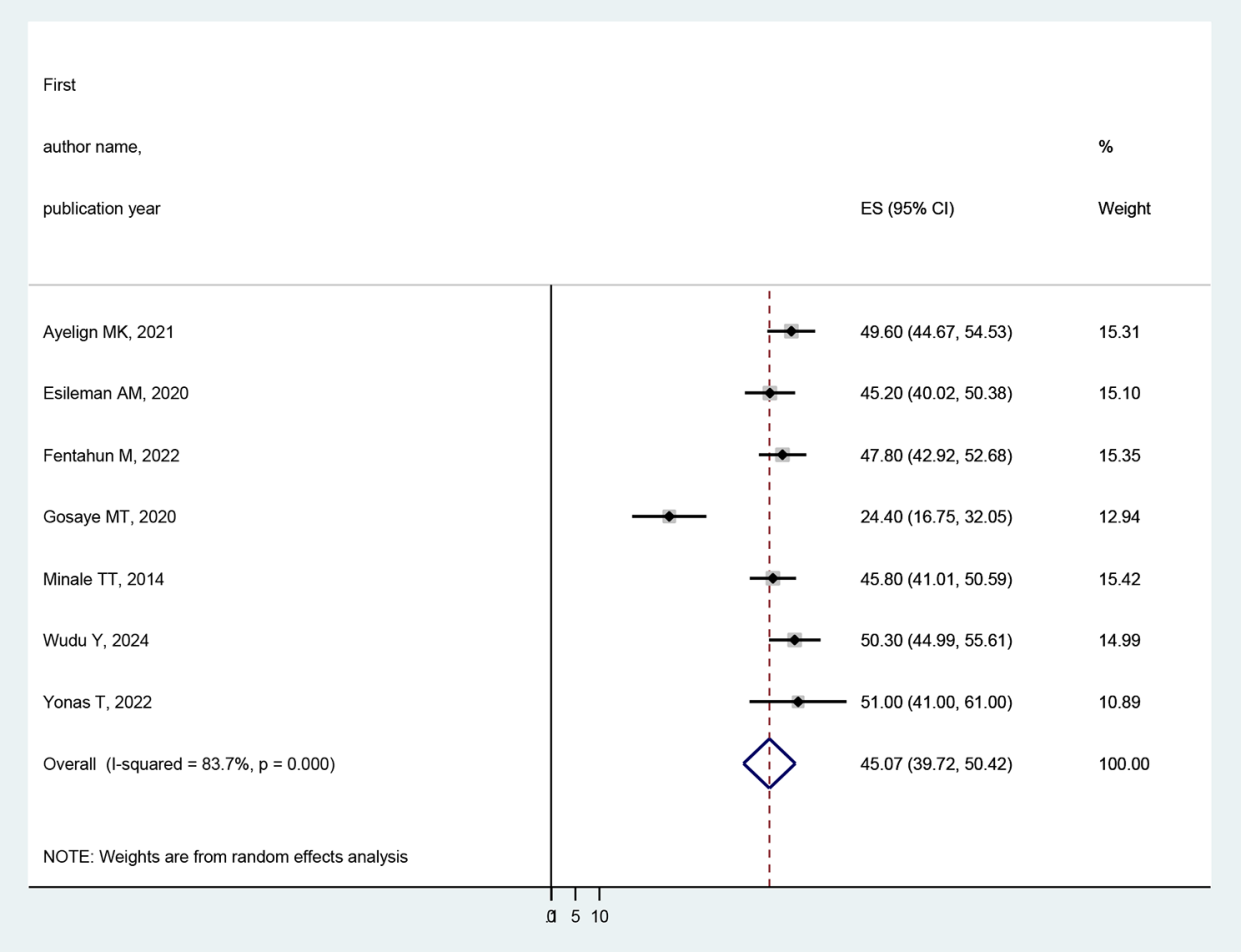
Forest plot of the pooled prevalence of poor quality of life among people with epilepsy in Ethiopia

Figure 2 shows that in this meta-analysis, seven studies were included to estimate the pooled prevalence of poor quality of life among PWE in Ethiopia. The prevalence of poor quality of life among the included studies shows the minimum and maximum results of 24.4% [23] and 51% [24], respectively. However, the result of the Gosaye MT et, al.2020 [23] study was different from the rest of the studies. The possible reason might be related to the small sample size. The pooled prevalence of poor quality of life among people with epilepsy in Ethiopia was 45.07 (95% CI: 39.73–50.42%). Based on the apparent heterogeneity among the studies, we conducted a meta-analysis using a random effect model (I2 = 83.7%, *p* < 0.001).

### Subgroup analysis of quality of life

**Fig. 3:**
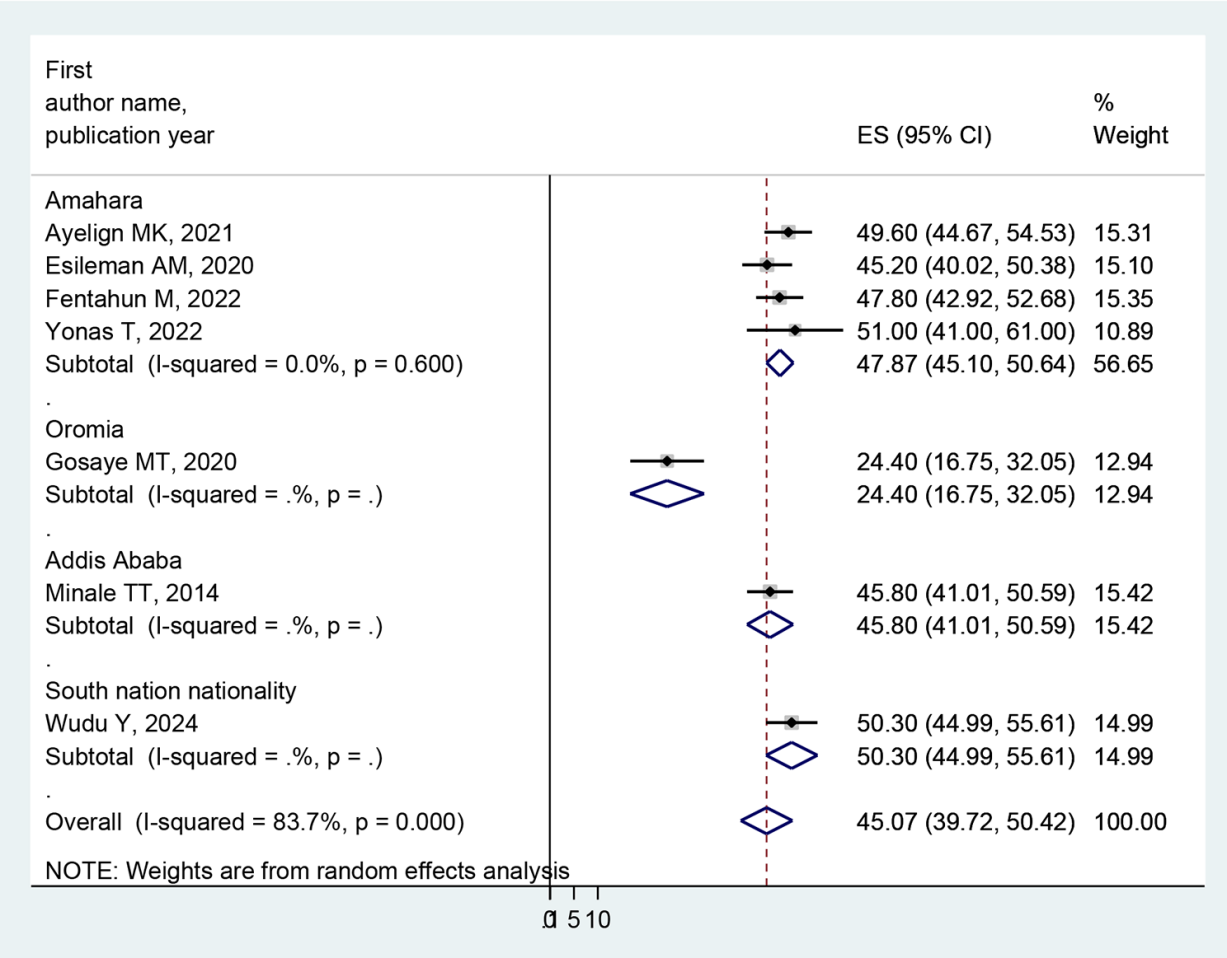
Forest plot, based on subgroup analysis based on the study region, of the pooled prevalence of poor quality of life among people with epilepsy in Ethiopia

**Fig. 4:**
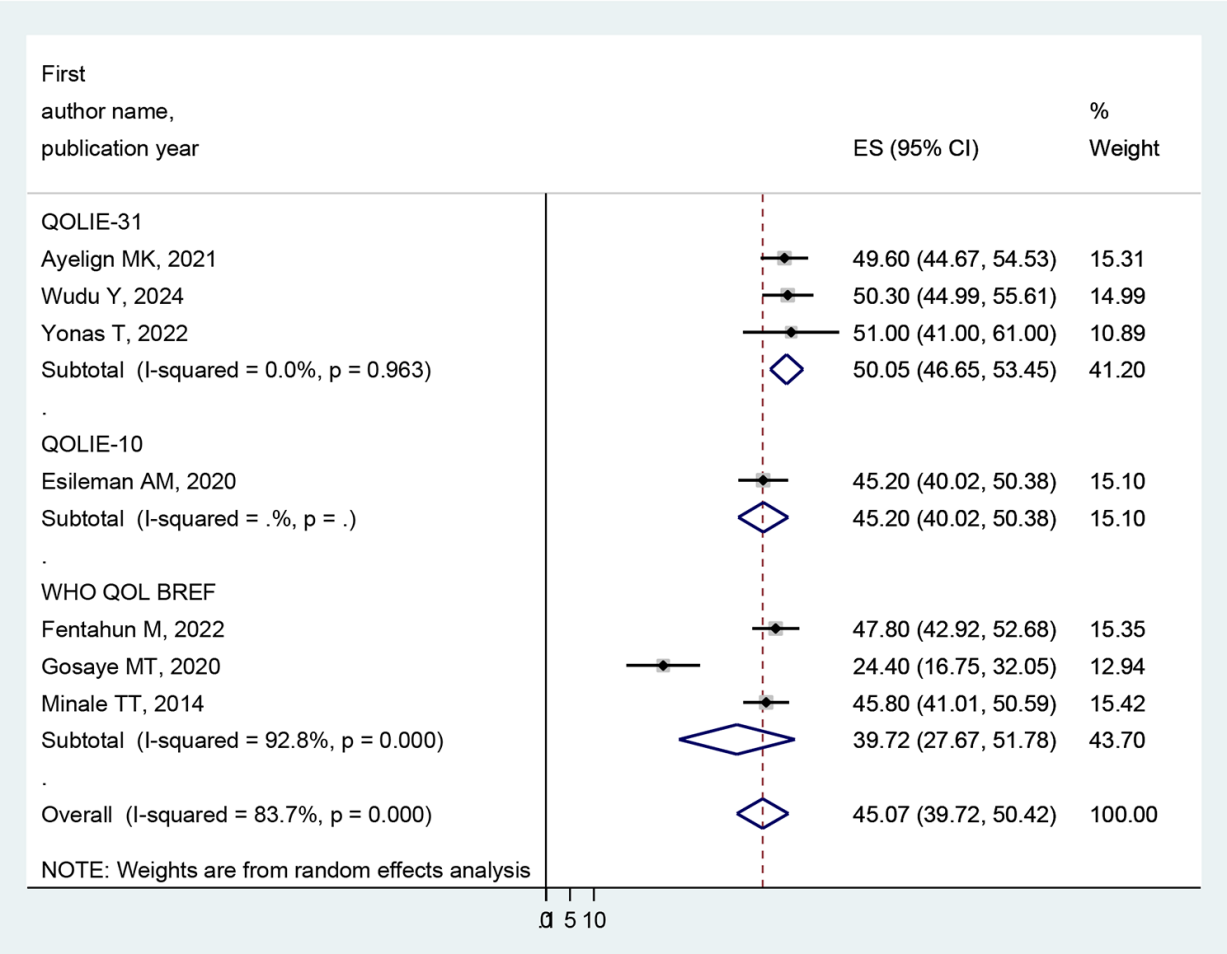
Forest plot, based on subgroup analysis based on the assessment tool, of the pooled prevalence of poor quality of life among people with epilepsy in Ethiopia

In a meta-analysis of the pooled prevalence of poor quality of life, heterogeneity was found (I^2^ = 83.7%, *p* < 0.001). Thus, a subgroup analysis was carried out on the study region and the assessment tool. The highest pooled prevalence of poor quality of life among people with epilepsy was found in Amara reginal state, 47.87% (95% CI: 45.10-50.64; I^2^ = 0.0%, *p* < 0.600) compared to the overall pooled prevalence. Regarding assessment tool of poor quality of life of people with epilepsy, QOLIE-31 accounted for 50.05% (95%CI: 46.65–53.45; I^2^ = 0.0%, *p* < 0.963), and WHO QOL BREF accounted for 39.72% (95%CI: 27.67–51.78; I^2^ = 92.8%, *p* < 0.001) (Figs. 3 and 4).

### Publication bias

**Fig. 5:**
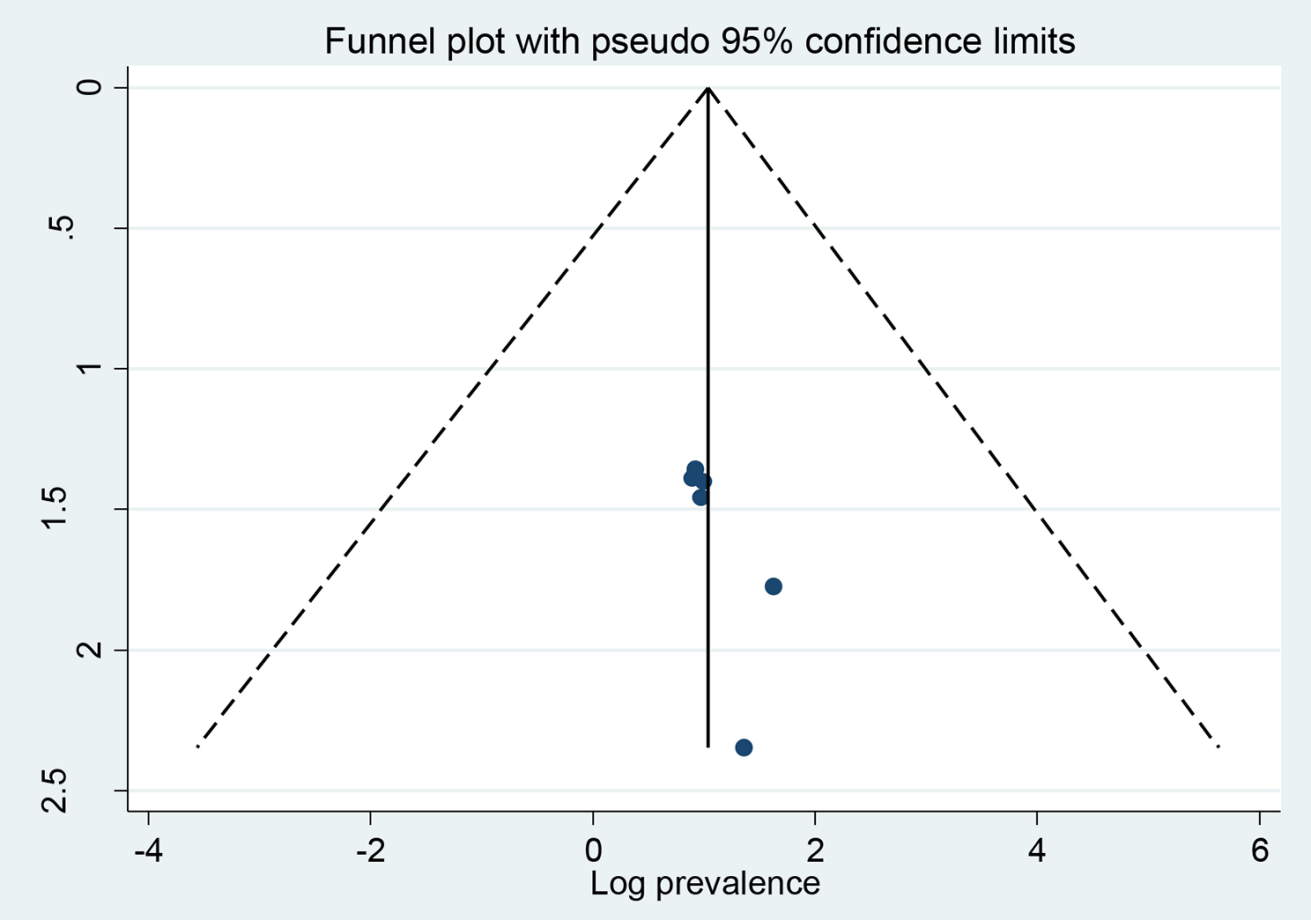
Funnel plot showing publication bias of the prevalence of poor quality of life among people with epilepsy in Ethiopia

The results of this study’s funnel plot (Fig. 5) show that there is no publication bias, and Egger’s regression test (*P* = 0.379) supported this finding (Table 2).

**Table 2:** Egger’s test of quality of life of people with epilepsy in Ethiopia

Std_−_Eff	Coef.	Std. Err.	T	*P* > t	[95% Conf Interval]	
Slope	57.55621	12.1769	4.73	0.005	26.25448	88.85793
Bias	-4.144217	4.290978	-0.97	0.379	-15.17453	6.886094

### A leave-out-one sensitivity analysis

**Fig. 6:**
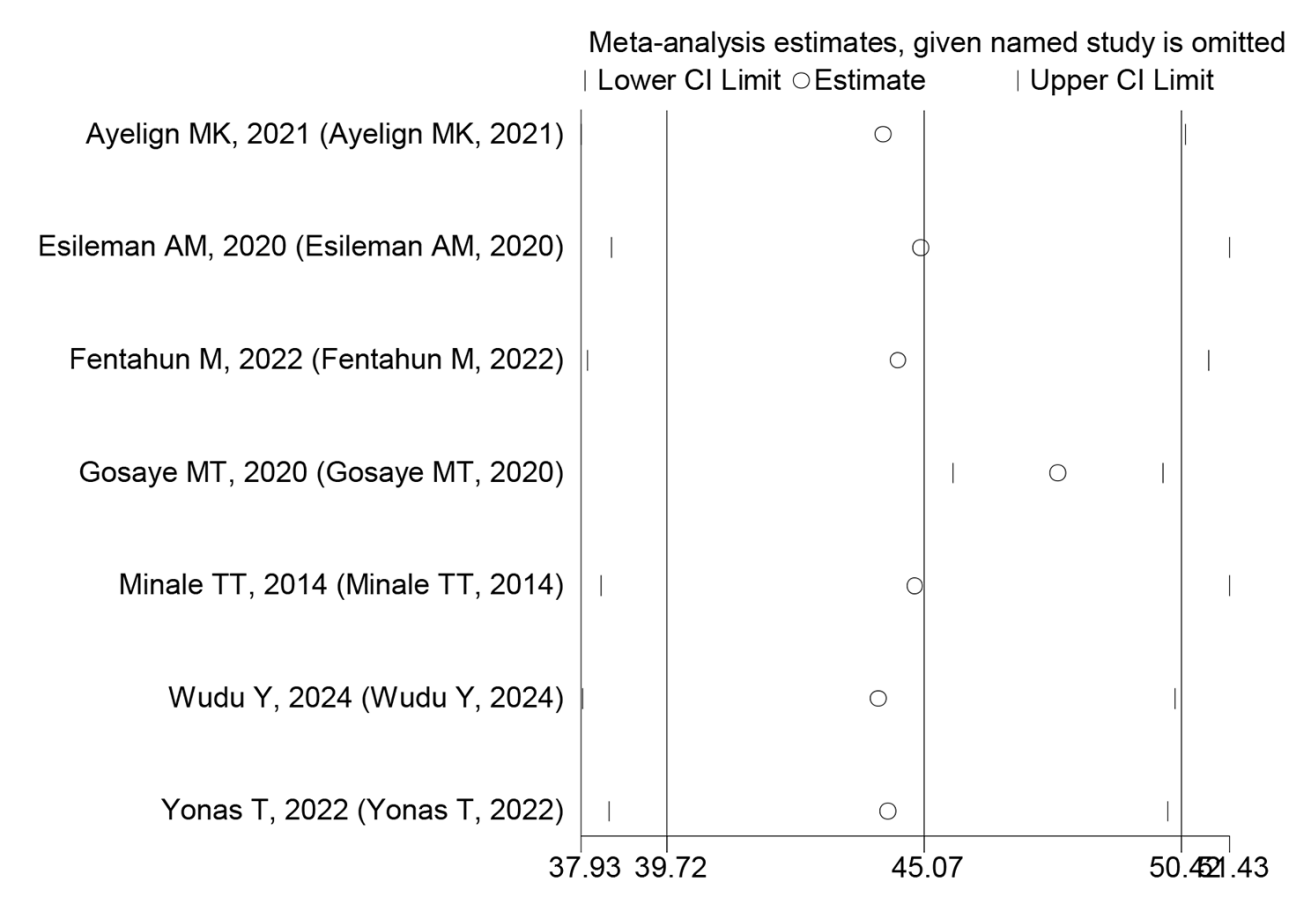
Sensitivity analysis of the prevalence of quality of life of people with epilepsy in Ethiopia, a study being removed at a time: prevalence and 95% CI (the analysis is based on a random effect model)

In this systematic review and meta-analysis study, the effect of each study on the pooled prevalence of poor quality of life among people with epilepsy was examined using a sensitivity analysis, which involved gradually eliminating one study at a time to test the heterogeneity of those findings. The prevalence of this systematic review and meta-analysis was not significantly affected by the omission of a single article, as shown by the results, which ranged from 42.22 to 47.85% (Table 3) and (Fig. 6).

**Table 3:** Sensitivity analysis of quality of life of people with epilepsy in Ethiopia

Study omitted	Estimated 95% CI	Heterogeneity	
		I^2^ (%)	*P*-value
Ayelign MK, 2021,	42.22(37.93–50.51)	85.4	0.000
Esileman AM, 2020	45.00(38.57–51.43)	86.3	0.000
Fentahun M, 2022	44.53(38.07-51.00)	86.2	0.000
Gosaye MT, 2020	47.85(45.67–50.04)	0.0	0.640
Minale TT, 2014	44.88(38.51.42)	86.4	0.000
Wudu Y, 2024	44.12(37.96–50.28)	85.2	0.000
Yonas T, 2022	44.32(38.51–50.14)	86	0.000

### Associated factors analysis

**Fig. 7:**
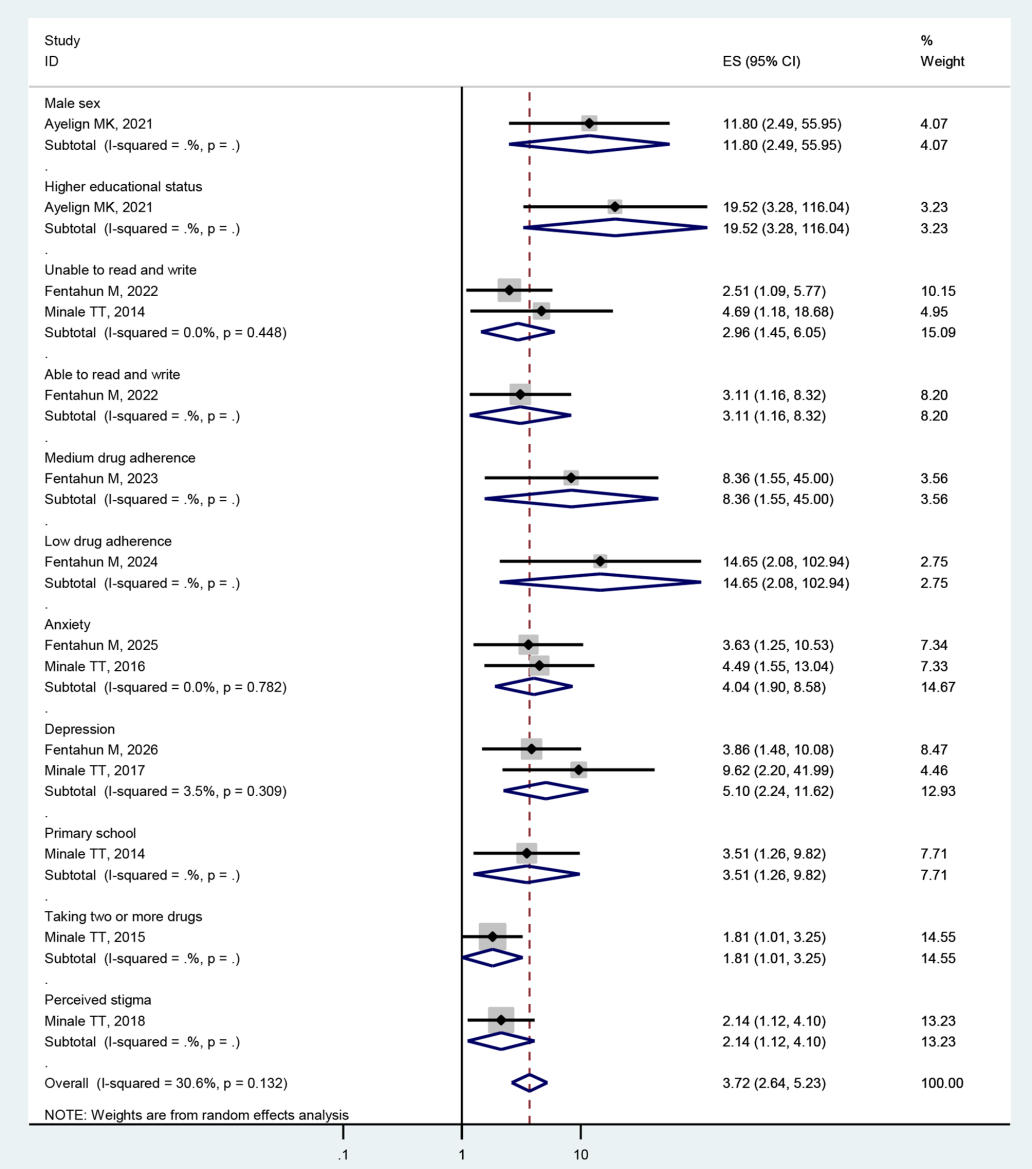
Forest plot showing a narrative synthesis of the findings regarding the associated factors

In Table 1, we extracted important sociodemographic and other factors that are associated with poor quality of life among PWE with reference to the studies analysed in logistic regression with an adjusted odd ratio. The pooled analysis was done to determine the pooled effect of the factors when the factors were associated with two or more papers. The individual papers found the following factors: being male, higher educational status [44], unable to read and write [50, 52], primary school [52], low drug adherence, medium drug adherence [50], taking two or more drugs [52], anxiety [50, 52], depression [50, 52], and perceived stigma [52]. Thus, in this review, in terms of demographic characteristics, being unable to read and write (AOR = 2.96, 95% CI: 1.45–6.05) was significantly associated with the quality of life of people with epilepsy. Regarding clinical factors, anxiety (AOR = 4.04, 95% CI: 1.90–8.58) and depression (AOR = 5.1, 95% CI: 2.24–11.62) were significantly linked to the quality of life of people with epilepsy (Fig. 7).

## Discussion

Patients with epilepsy have a lower quality of life than people without epilepsy. When their epilepsy is under control, their quality of life is either comparable to or worse than that of patients with other chronic diseases [56]. The prevalence of female epileptic patients was lower than that of male epileptic patients, and a higher quality of life score [57] was obtained by married individuals. Ethiopia is a developing nation where epilepsy was shown to be more common in those with lower socioeconomic position [6].

In this review, we have included 7 studies conducted in Ethiopia with 2123 study participants, of whom 1163 (54.78%) were male individuals and 1196 (56.34%) were living without marriage (either single, divorced, or widowed). The analysis of the pooled prevalence of poor quality of life among people with epilepsy in Ethiopia is 45.07 (95% CI: 39.73–50.42%). This finding was consistent with previous studies conducted on the quality of life among PWE in Kenya [22], and Nigeria [26]; poor quality of life among PWE in both countries was 49.90%, and 44.6%, respectively. Whereas it was higher than with a previous study on quality of life among PWE conducted in Lebanese [21], the study showed that the poor QoL was 38.6% of PWE. This discrepancy may be caused by differences in the residence of study participants, the sample size, and the measurement tool. This analysis included seven studies of 2123 Ethiopians who live with epilepsy in Ethiopia, using the following assessment tools: the 31 items in a QOLIE-31 questionnaire [53], the 26 items of the general WHOQOL-BREF [54], and the QOLIE-10 questionnaire [55]. In contrast, 404 research participants in the prior study, which was done in Lebanon, were evaluated using the QOLIE-10 [21].

In the current study, regarding subgroup analysis, the highest pooled prevalence of poor quality of life among people with epilepsy was found in Amara regional state (47.87%) compared to the overall pooled prevalence. This finding may vary depending on the number of studies, participant sample size, and assessment tool. Regarding the assessment tool of poor quality of life for people with epilepsy, QOLIE-31 accounted for 50.05%, and WHO QOL BREF accounted for 39.72%. This discrepancy might be due to the number of items and their domains [53, 54].

According to our review, anxiety, depression, and being unable to read or write had a significant association with the quality of life of PWE. In the previous studies, having mental comorbidities such as anxiety and depression and having a deficient understanding of epilepsy [21] were significant predictors of the poor quality of life of PWE [21, 26, 27, 56]. Additionally, the varied QoL of these patients across nations is associated with their clinical presentation, socioeconomic status, and demographic traits [6]. Thus, our results supported the significant relationship between clinical aspects (anxiety and depression), demographic traits (unable to read and write), and the quality of PWE. Epilepsy significantly affects PWE’s physical and mental well-being, interfering with their daily and occupational activities and overall quality of life. The condition’s management may also worsen PWE’s quality of life. There may be a lack of awareness regarding the effects of epilepsy on mental health and mood [58]. In order to minimise the challenges faced by individuals with epilepsy as well as poor quality of life, stakeholders (healthcare policy-makers or clinical practitioners) may find this finding useful in addressing the prevention, early screening, and management of individuals with epilepsy. More representative samples, or rather, a cross-sectional study design, should be used in future studies aiming for a more precise diagnosis.

### Strengths and limitations of the study

New insights into managing and enhancing the quality of life for individuals with epilepsy could result from this research. The fact that this study only covered a small number of papers that were assessed using different measurement tools, only included research that was published in English, and missed a crucial factor in quality of life such as stigma were its limitations. It was also difficult to appraise and describe the results because most studies used rather diverse instruments to measure quality of life.

## Conclusion

This review found that there was a high pooled prevalence of poor quality of life related to people with epilepsy in Ethiopia. Demographic characteristics (unable to read and write), anxiety, and depression were risk factors for poor quality of life among PWE. This study may provide further information to concerned bodies that do early screening and manage the quality of life of individuals with epilepsy. Also, screening and intervention of anxiety and depression problems should be considered in regular epilepsy care management.

### Electronic supplementary material

Below is the link to the electronic supplementary material.

Supplementary Material 1

Supplementary Material 2

Supplementary Material 3

## Data Availability

This published article and its supplementary information files include all data generated or analysed during this study.

## References

[1] Sadock BJ. Kaplan & Sadock’s synopsis of psychiatry: behavioral sciences/clinical psychiatry. Wolters Kluwer Philadelphia; 2015.

[2] Falco-Walter JJ, Scheffer IE, Fisher RS (2018). The new definition and classification of seizures and epilepsy. Epilepsy Res.

[3] Ngugi AK, Bottomley C, Kleinschmidt I, Sander JW, Newton CR (2010). Estimation of the burden of active and life-time epilepsy: a meta‐analytic approach. Epilepsia.

[4] Beghi E, Giussani G, Nichols E, Abd-Allah F, Abdela J, Abdelalim A (2019). Global, regional, and national burden of epilepsy, 1990–2016: a systematic analysis for the global burden of Disease Study 2016. Lancet Neurol.

[5] Mbizvo GK, Bennett K, Simpson CR, Duncan SE, Chin RF (2022). Epilepsy-related causes of mortality in people with epilepsy: a systematic review of systematic reviews. J Neurol Neurosurg Psychiatry.

[6] Sadr SS, Javanbakht J, Javidan AN, Ghaffarpour M, Khamse S, Naghshband Z (2018). Descriptive epidemiology: prevalence, incidence, sociodemographic factors, socioeconomic domains, and quality of life of epilepsy: an update and systematic review. Archives Med Sci.

[7] Kerr MP (2012). The impact of epilepsy on patients’ lives. Acta Neurol Scand.

[8] Ghanean H, Nojomi M, Jacobsson L (2013). Public awareness and attitudes towards epilepsy in Tehran. Iran Global Health Action.

[9] Kubota H, Awaya Y (2010). Assessment of health-related quality of life and influencing factors using QOLIE-31 in Japanese patients with epilepsy. Epilepsy Behav.

[10] Perucca P, Gilliam FG, Schmitz B (2009). Epilepsy treatment as a predeterminant of psychosocial ill health. Epilepsy Behav.

[11] Akosile CO, Anomneze JU, Okoye EC, Adegoke BOA, Uwakwe R, Okeke E (2021). Quality of life, fatigue and seizure severity in people living with epilepsy in a selected Nigerian population. Seizure.

[12] Baker GA. The psychosocial burden of epilepsy. 2002. 10.1046/j.1528-1157.43.s.6.12.x 12190975

[13] Quintas R, Raggi A, Giovannetti AM, Pagani M, Sabariego C, Cieza A (2012). Psychosocial difficulties in people with epilepsy: a systematic review of literature from 2005 until 2010. Epilepsy Behav.

[14] Ferrans CE, editor. Editor quality of life: conceptual issues. Seminars in oncology nursing; 1990. 10.1016/0749-2081(90)90026-2 2274721

[15] Bowling A, Brazier J, Introduction-quality-of-life in social-science, and medicine. Pergamon-elsevier science ltd the boulevard, langford lane, kidlington … p. 1337–8.

[16] Peasgood T, Brazier J, Mukuria C, Rowen D. A conceptual comparison of well-being measures used in the UK. 2014.

[17] Rejeski, Mihalko (2001). Physical activity and quality of life in older adults. Journals Gerontol Ser A: Biol Sci Med Sci.

[18] Cummins RA (2005). Moving from the quality of life concept to a theory. J Intellect Disabil Res.

[19] Felce D, Perry J (1995). Quality of life: its definition and measurement. Res Dev Disabil.

[20] Meeberg GA (1993). Quality of life: a concept analysis. J Adv Nurs.

[21] Tarhini Z, Jost J, Ratsimbazafy V, Preux P-M, Salameh P, Al-Hajje A (2022). Knowledge of epilepsy, quality of life, and psychiatric comorbidities in Lebanese adults with epilepsy. Epilepsy Behav.

[22] Kinyanjui DW, Kathuku DM, Mburu JM (2013). Quality of life among patients living with epilepsy attending the neurology clinic at Kenyatta National Hospital, Nairobi, Kenya: a comparative study. Health Qual Life Outcomes.

[23] Tefera GM, Megersa WA, Gadisa DA (2020). Health-related quality of life and its determinants among ambulatory patients with epilepsy at Ambo General Hospital, Ethiopia: using WHOQOL-BREF. PLoS ONE.

[24] Teshome Y, Solomon Y, Talargia F, Worku N, Shitaw A, Leminie AA. Level of Acceptance of Illness and Its Association with Quality of Life among Patients with Epilepsy in North Shewa, Ethiopia. Behavioural Neurology. 2022;2022. 10.1155/2022/1142215 PMC9484932 36134035

[25] Baranowski CJ (2018). The quality of life of older adults with epilepsy: a systematic review. Seizure.

[26] Ayanda KA, Sulyman D (2020). Determinants of quality of life in adults living with epilepsy. Ann Afr Med.

[27] Tsigebrhan R, Derese A, Kariuki SM, Fekadu A, Medhin G, Newton CR (2023). Co-morbid mental health conditions in people with epilepsy and association with quality of life in low-and middle-income countries: a systematic review and meta-analysis. Health Qual Life Outcomes.

[28] Chaka A, Awoke T, Yohannis Z, Ayano G, Tareke M, Abate A (2018). Determinants of depression among people with epilepsy in Central Ethiopia. Ann Gen Psychiatry.

[29] Alemu WG, Due C, Muir-Cochrane E, Mwanri L, Azale T, Ziersch A. Quality of life among people living with mental illness and predictors in Africa: a systematic review and meta-analysis. Qual Life Res. 2023:1–19. 10.1007/s11136-023-03525-8 PMC11045618 37906348

[30] Page MJ, McKenzie JE, Bossuyt PM, Boutron I, Hoffmann TC, Mulrow CD (2021). The PRISMA 2020 statement: an updated guideline for reporting systematic reviews. Int J Surg.

[31] Stroup DF, Berlin JA, Morton SC, Olkin I, Williamson GD, Rennie D (2000). Meta-analysis of observational studies in epidemiology: a proposal for reporting. Meta-analysis of Observational studies in Epidemiology (MOOSE) group. JAMA.

[32] Hosseini M-S, Jahanshahlou F, Akbarzadeh M, Zarei M, Vaez-Gharamaleki Y. Formulating research questions for evidence-based studies. J Med Surg Public Health. 2023:100046.

[33] Munn Z, Moola S, Lisy K, Riitano D, Tufanaru C (2015). Methodological guidance for systematic reviews of observational epidemiological studies reporting prevalence and cumulative incidence data. Int J Evid Based Healthc.

[34] DerSimonian R, Laird N (2015). Meta-analysis in clinical trials revisited. Contemp Clin Trials.

[35] Huedo-Medina TB, Sánchez-Meca J, Marín-Martínez F, Botella J (2006). Assessing heterogeneity in meta-analysis: Q statistic or I2 index?. Psychol Methods.

[36] Lee YH (2018). Overview of the process of conducting meta-analyses of the diagnostic test accuracy. J Rheumatic Dis.

[37] Sterne JA, Egger M (2001). Funnel plots for detecting bias in meta-analysis: guidelines on choice of axis. J Clin Epidemiol.

[38] Egger M, Davey Smith G, Schneider M, Minder C (1997). Bias in meta-analysis detected by a simple, graphical test. BMJ.

[39] Gebre AK, Haylay A, Sociodemographic (2018). Clinical variables, and quality of life in patients with Epilepsy in Mekelle City, Northern Ethiopia. Behav Neurol.

[40] Tsigebrhan R, Fekadu A, Medhin G, Newton CR, Prince MJ, Hanlon C (2021). Comorbid mental disorders and quality of life of people with epilepsy attending primary health care clinics in rural Ethiopia. PLoS ONE.

[41] Alemu A, Dendir G, Gonfa A, Sisay Y, Tadesse T, Abebe A (2023). Health-related quality of life and associated factors among adult patients with epilepsy in public hospitals of Wolaita Zone, southern Ethiopia. An embedded mixed method study. Epilepsy Behav.

[42] Addis B, Minyihun A, Aschalew AY (2021). Health-related quality of life and associated factors among patients with epilepsy at the University of Gondar comprehensive specialized hospital, northwest Ethiopia. Qual Life Res.

[43] Mesafint G, Shumet S, Habtamu Y, Fanta T, Molla G (2020). Quality of Life and Associated factors among patients with Epilepsy Attending Outpatient Department of Saint Amanuel Mental Specialized Hospital, Addis Ababa, Ethiopia, 2019. J Multidiscip Healthc.

[44] Kassie AM, Abate BB, Kassaw MW, Getie A, Wondmieneh A, Tegegne KM (2021). Quality of life and its associated factors among epileptic patients attending public hospitals in North Wollo Zone, Northeast Ethiopia: a cross-sectional study. PLoS ONE.

[45] Alemayehu DS, Hailu E. Quality of Life Assessment among adult epileptic patients taking follow up care at Jimma University Medical Center, Jimma, South West Ethiopia: using quality of life in Epilepsy Inventory-31instrument. The Journal of medical research; 2018.

[46] Abadiga M, Mosisa G, Amente T, Oluma A (2019). Health-related quality of life and associated factors among epileptic patients on treatment follow up at public hospitals of wollega zones, Ethiopia, 2018. BMC Res Notes.

[47] Stotaw AS, Kumar P, Beyene DA, Tadesse TA, Abiye AA (2022). Health-related quality of life and its predictors among people living with epilepsy at Dessie Referral Hospital, Amhara, Ethiopia: a cross-sectional study. SAGE Open Med.

[48] Muche E, Ayalew M. Quality of life of epileptic patients in Resource Limited setting. SSRN Electronic Journal; 2018.

[49] Muche EA, Ayalew MB, Abdela OA. Assessment of quality of life of epileptic patients in Ethiopia. International Journal of Chronic Diseases. 2020;2020. 10.1155/2020/8714768 PMC6961609 31976314

[50] Minwuyelet F, Mulugeta H, Tsegaye D, Lake B, Getie A, Tsegaye B et al. Quality of life and associated factors among patients with epilepsy at specialized hospitals, Northwest Ethiopia; 2019. PLoS One. 2022;17(1):e0262814. 10.1371/journal.pone.0262814 PMC8794165 35085331

[51] Yesuf W, Hiko D, Alemayehu E, Kusheta S, Shita A, Beyene M (2024). Health-related quality of life in epilepsy and its associated factors among adult patients with epilepsy attending Mizan Tepi University Teaching Hospital, Southwest Ethiopia: a cross-sectional study. BMJ open.

[52] Tegegne MT, Muluneh NY, Wochamo TT, Awoke AA, Mossie TB, Yesigat MA (2014). Assessment of quality of life and associated factors among people with epilepsy attending at Amanuel Mental Specialized Hospital, Addis Ababa, Ethiopia. Science.

[53] Devinsky O, Cramer J, the QOLIE Development Group. Professional Postgraduate Services, a division of Physicians World Communications Group, Secaucus, NJ, USA. 1993.

[54] Organization WH. WHOQOL-BREF: introduction, administration, scoring and generic version of the assessment: field trial version, December 1996. World Health Organization; 1996.

[55] Cramer JA, Perrine K, Devinsky O, Meador K (1996). A brief questionnaire to screen for quality of life in epilepsy the QOLIE-10. Epilepsia.

[56] Berto P (2002). Quality of life in patients with epilepsy and impact of treatments. PharmacoEconomics.

[57] Shetty PH, Punith K, Naik RK, Saroja A (2011). Quality of life in patients with epilepsy in India. J Neurosciences Rural Pract.

[58] Strzelczyk A, Aledo-Serrano A, Coppola A, Didelot A, Bates E, Sainz-Fuertes R (2023). The impact of epilepsy on quality of life: findings from a European survey. Epilepsy Behav.

