# Supplementary material for: Quality of life and associated factors among people with epilepsy in Ethiopia: a systematic review and meta-analysis
Source: BMC Public Health. 2024 Jun 7;24:1529. doi: 10.1186/s12889-024-19018-3 (PMC11157882; doi:10.1186/s12889-024-19018-3)
Supplement: Supplementary file 2 — Supplementary Material 2 [file 12889_2024_19018_MOESM2_ESM.docx]

**Database searching strategies:**

**Cochrane library**

1241 Cochrane Reviews matching (prevalence OR epidemiology OR magnitude AND "quality of life" OR QoL AND “associated factors” OR predictors AND "people with epilepsy" OR “patients with epilepsy” AND Ethiopia) in Title Abstract Keyword

**EMBASE**

(((('prevalence' OR 'epidemiology' OR 'magnitude') AND 'quality of life' OR 'qol') AND 'associated factors' OR 'predictors') AND 'people with epilepsy' OR 'patients with epilepsy') AND 'ethiopia' AND [embase]/lim

**MEDLINE**

(((('prevalence' OR 'epidemiology' OR 'magnitude') AND 'quality of life' OR 'qol') AND 'associated factors' OR 'predictors') AND 'people with epilepsy' OR 'patients with epilepsy') AND 'ethiopia' AND [medline]/lim
