# Supplementary material for: Quality of life and associated factors among people with epilepsy in Ethiopia: a systematic review and meta-analysis
Source: BMC Public Health. 2024 Jun 7;24:1529. doi: 10.1186/s12889-024-19018-3 (PMC11157882; doi:10.1186/s12889-024-19018-3)
Supplement: Supplementary file 3 — Supplementary Material 3 [file 12889_2024_19018_MOESM3_ESM.docx]

Quality assessment

| First author name (year) | Q1 | Q2 | Q3 | Q4 | Q5 | Q6 | Q7 | Q8 | Q9 | Total score (9%) |
| --- | --- | --- | --- | --- | --- | --- | --- | --- | --- | --- |
| Ayelign MK, 2021 | NA | Y | NR | Y | Y | Y | Y | Y | Y | 8 |
| Esileman AM, 2020 | NA | Y | Y | Y | Y | Y | Y | Y | Y | 8 |
| Fentahun M, 2022 | NA | Y | Y | Y | Y | Y | Y | Y | Y | 8 |
| Gosaye MT, 2020 | NA | Y | NA | Y | Y | Y | Y | Y | Y | 7 |
| Minale TT, 2014 | NA | Y | NR | Y | Y | Y | Y | Y | Y | 7 |
| Wudu Y, 2024 | Y | Y | Y | Y | Y | Y | Y | Y | NR | 8 |
| Yonas T, 2022 | NR | NR | NR | Y | Y | Y | Y | Y | Y | 6 |

**Key:** **Y**= Yes; **N**= No; **NR**= Not Reported, **NA**=Not Applicable

**Question codes:**

1. Was the sample frame appropriate to address the target population?

2. Were study participants sampled in an appropriate way?

3. Was the sample size adequate?

4. Were the study subjects and the setting described in detail?

5. Was the data analysis conducted with sufficient coverage of the identified sample?

6. Were valid methods used for the identification of the condition?

7. Was the condition measured in a standard, reliable way for all participants?

8. Was there appropriate statistical analysis?

9. was the response rate adequate, and if not, was the low response rate managed appropriately?
